# EGR1 Upregulation during Encephalitic Viral Infections Contributes to Inflammation and Cell Death

**DOI:** 10.3390/v14061210

**Published:** 2022-06-02

**Authors:** Caitlin W. Lehman, Amy Smith, Jamie Kelly, Jonathan L. Jacobs, Jonathan D. Dinman, Kylene Kehn-Hall

**Affiliations:** 1 Department of Biomedical Sciences and Pathobiology, Virginia-Maryland College of Veterinary Medicine, Virginia Polytechnic Institute and State University, Blacksburg, VA 24061, USA; woodsonc@vt.edu (C.W.L.); amysmith104@gmail.com (A.S.); 2 Center for Emerging, Zoonotic, and Arthropod-Borne Pathogens, Virginia Polytechnic Institute and State University, Blacksburg, VA 24061, USA; 3 Department of Cell Biology and Molecular Genetics, University of Maryland, College Park, MD 20742, USA; jkelly22@umd.edu (J.K.); dinman@umd.edu (J.D.D.); 4 American Type Culture Collection, Manassas, VA 20110, USA; jjacobs@atcc.org

**Keywords:** Venezuelan equine encephalitis, astrocytes, EGR1, inflammation, alphaviruses

## Abstract

Early growth response 1 (EGR1) is an immediate early gene and transcription factor previously found to be significantly upregulated in human astrocytoma cells infected with Venezuelan equine encephalitis virus (VEEV). The loss of EGR1 resulted in decreased cell death but had no significant impact on viral replication. Here, we extend these studies to determine the impacts of EGR1 on gene expression following viral infection. Inflammatory genes CXCL3, CXCL8, CXCL10, TNF, and PTGS2 were upregulated in VEEV-infected cells, which was partially dependent on EGR1. Additionally, transcription factors, including EGR1 itself, as well as ATF3, FOS, JUN, KLF4, EGR2, and EGR4 were found to be partially transcriptionally dependent on EGR1. We also examined the role of EGR1 and the changes in gene expression in response to infection with other alphaviruses, including eastern equine encephalitis virus (EEEV), Sindbis virus (SINV), and chikungunya virus (CHIKV), as well as Zika virus (ZIKV) and Rift Valley fever virus (RVFV), members of the *Flaviviridae* and *Phenuiviridae* families, respectively. EGR1 was significantly upregulated to varying degrees in EEEV-, CHIKV-, RVFV-, SINV-, and ZIKV-infected astrocytoma cells. Genes that were identified as being partially transcriptionally dependent on EGR1 in infected cells included ATF3 (EEEV, CHIKV, ZIKV), JUN (EEEV), KLF4 (SINV, ZIKV, RVFV), CXCL3 (EEEV, CHIKV, ZIKV), CXCL8 (EEEV, CHIKV, ZIKV, RVFV), CXCL10 (EEEV, RVFV), TNF-α (EEEV, ZIKV, RVFV), and PTGS2 (EEEV, CHIKV, ZIKV). Additionally, inhibition of the inflammatory gene PTGS2 with Celecoxib, a small molecule inhibitor, rescued astrocytoma cells from VEEV-induced cell death but had no impact on viral titers. Collectively, these results suggest that EGR1 induction following viral infection stimulates multiple inflammatory mediators. Managing inflammation and cell death in response to viral infection is of utmost importance, especially during VEEV infection where survivors are at-risk for neurological sequalae.

## 1. Introduction

Alphaviruses belong to the viral family *Togaviridae* and are further subdivided into either Old World or New World alphaviruses based on their genetic sequence and the type of clinical disease they cause [1]. Old World alphaviruses, including Sindbis virus (SINV) and chikungunya virus (CHIKV), induce arthritic disease and are primarily endemic to Africa, Asia, Europe, Australia, and Central and South America [2]. Although not as common, both CHIKV and SINV can also cause neurological disease [3]. Meanwhile, New World alphaviruses, such as Venezuelan equine encephalitis virus (VEEV) and eastern equine encephalitis virus (EEEV), induce encephalitic disease and are endemic to the Americas [2]. As zoonotic pathogens primarily propagated by mosquito vectors, these viruses are capable of causing massive disruption in both human and animal health. VEEV and EEEV are of particular concern because they are neurotropic viruses capable of causing detrimental damage to the central nervous system (CNS) and survivors are often afflicted with lifelong neurological sequalae [4]. Currently, there are no FDA-approved vaccines or therapeutic interventions available and, as such, a better understanding of the molecular underpinnings of these viruses is urgently needed. 

Previous transcriptomic studies identified the immediate early gene (IEG) and transcription factor, early growth response 1 (EGR1), as being highly upregulated in U87MG astrocytoma cells in response to VEEV infection [5]. EGR1 is a Cys2-His2-type zinc-finger transcription factor that is activated by a wide array of stimuli, such as growth factors, cytokines, apoptosis, and cellular stress states [6]. Upon induction, EGR1 functions as a convergence point for numerous specialized signaling cascades and couples short-term extracellular signals with the transcriptional regulation of genes required to initiate the appropriate biological response [7]. In addition to EGR1, the early growth response (EGR) family also includes EGR2, EGR3, and EGR4. All four members of the EGR family share high sequence homology, especially within the DNA binding domains, thus suggesting a possible functional overlap among family members [6]. 

EGR1 has been implicated in the regulation of inflammation associated with hepatic injury [8], lung injury [9], and atherogenesis [10]. Several mechanisms by which EGR1 regulates inflammation have been proposed and are likely specific to the species and cell type as well as the insult encountered. EGR1 is a positive regulator of B- and T-cell-mediated responses through transcriptional control of cytokines and costimulatory molecules [11]. Additionally, EGR1 can indirectly interact with the nucleosome remodeling and deacetylation corepressor complex to reduce chromatin accessibility through deacetylation in order to suppress inflammatory genes [12]. EGR1 has also been implicated in apoptosis, most notably in cancer studies where EGR1 expression is significantly reduced in developing tumors [13]. Upon exposure to damaging ionizing radiation, EGR1 binds to PTEN, a tumor suppressor and pro-apoptotic gene, to induce apoptosis [14]. Another pro-apoptotic gene, the NSAID-activated gene 1 (NAD-1), is regulated by EGR1 and has been shown to facilitate apoptosis in colon carcinoma cells, lung cancer cells, and hepatocellular carcinoma cells [15,16]. 

The role of EGR1 in viral infections has been explored by our lab and others. EGR1 upregulation has been shown to be critical in Epstein–Barr virus (EBV) infection [17]. In Kaposi’s sarcoma-associated herpesvirus (KSHV), EGR1 has been shown to be critically involved in latent virus reactivation [18]. Further, the disruption of EGR1 binding to the viral genome prevented the establishment of viral latency in human cytomegalovirus (CMV) infections [19]. EGR1 has also been identified to play an antiviral role in foot-and-mouth disease virus (FMDV) replication [20]. In our studies involving EGR1, we have shown that EGR1 is upregulated in VEEV-infected astrocytes, and the loss of EGR1 results in decreased VEEV-induced cell death. EGR1 was also identified as a novel link between two pathways intimately associated with apoptosis: the interferon response pathway and the unfolded protein response pathway [5]. Additionally, we found that EGR1 upregulation and the accompanying cell death following VEEV infection is modulated by ERK and PERK pathways [21]. Here, we further elucidate the influence of EGR1 on the gene expression of inflammatory markers and apoptotic transcriptional machinery following viral infection. 

## 2. Materials and Methods

### 2.1. Identification of Candidate EGR1 Target Genes

Differentially expressed genes (DEGs) during VEEV infection of U87MG cells described previously [5] were studied with Ingenuity Pathway Analysis (IPA) [22]. Activation of EGR1 was confirmed by a right-tailed Fisher’s Exact Test for over-representation of downstream EGR1 targets in the list of DEGs. These EGR1 target genes were further interrogated for connectivity in the Ingenuity knowledge base to biological functions of neuronal cell death, inflammation, and encephalitis. Additional upstream regulators of the DEGs were identified. Promoter regions ±1kb of the TSS of all downstream EGR1 target genes and upstream regulators of the DEG list were searched for enrichment of the EGR1 transcription factor binding site using the TRANSFAC database [23]. 

### 2.2. Cell Culture

U87MG (ATCC HTB-14) and Vero (ATCC CCL-81) cells were obtained from ATCC (Manassas, VA, USA). EGR1−/− U87MG cells were generated as described below. All cells were maintained at 37 °C and 5% CO_2_. Culture medium consisted of Dulbecco’s modified minimum essential medium (DMEM; VWR, 10128-206) supplemented with 10% fetal bovine serum (FBS; VWR, 97068-085), 1% L-Glutamine (VWR, 45000-676), and 1% penicillin/streptomycin (VWR, 45000-652). 

### 2.3. Viruses and Viral Infections

VEEV TC-83 and VEEV Trinidad Donkey (TrD) (GenBank Accession Number L01442.2) viral stocks were produced by electroporation of BHK-J cells with in vitro-transcribed viral RNA generated from molecular clones as described previously [24]. Rift Valley fever virus (RVFV) MP-12 was rescued using a reverse genetics system as previously described [25]. Zika virus (ZIKV) MR766 was obtained from BEI Resources (Manassas, VA, USA), catalog number NR-50065. CHIKV (181/25), live attenuated vaccine strain, was obtained from Dr. Naomi Forrester, University of Texas Medical Branch, Galveston, TX, USA. SINV (EgAr 339) was obtained from BEI Resources (NR-15695). Eastern equine encephalitis virus (EEEV) strain GA97 stocks were generated as previously described [26]. Experiments with VEEV TC-83, RVFV MP-12, ZIKV, CHIKV, and SINV were performed under BSL-2 conditions. Experiments with VEEV TrD, EEEV GA97, and RVFV ZH-548 were performed under BSL-3 conditions. Work involving select agents was conducted at Virginia Polytechnic Institute and State University (Virginia Tech) Infectious Disease Unit which is registered with the Center for Disease Control and Prevention (CDC). All work was performed in accordance with federal select agent regulations. 

For viral infections, cells were plated in 12-well cell culture-treated plates at a density of 1 × 10^5^ cells per well and incubated overnight at 37 °C and 5% CO_2._ The following day, cells were infected at the specified multiplicity of infection (MOI). After 1 h incubation with viral inoculum, the cells were washed twice with phosphate-buffered saline (PBS), and fresh medium was added back to the cells. Viral supernatants and cell lysates were collected at various times post-infection for further analysis. 

### 2.4. Western Blot Analysis

Cells were lysed in a blue lysis buffer composed of 25 mL 2× Novex Tris-Glycine Sample Loading Buffer SDS (Invitrogen, LC2676, Waltham, MA, USA), 20 mL T-PER Tissue Protein Extraction Reagent (Thermo Fisher Scientific, 78510, Waltham, MA, USA), 200 µL 0.5 M EDTA pH 8.0, 80 µL 0.1 M Na_3_VO_4_, 400 µL 0.1 M NaF, 1.3 mL 1 M dithiothreitol and 3-cOmplete Protease Inhibitor Cocktail tablets (Millipore Sigma, 11836170001, Burlington, MA, USA). A total of 25 µL of cell lysate was separated by gel electrophoresis on a NuPAGE 4–12% Bis-Tris gel (Invitrogen, NP0322BOX, Waltham, MA, USA) and then transferred to a PVDF membrane (Thermo Fisher Scientific, 88518, Waltham, MA, USA). Next, the membrane was blocked using 5% milk in tris-buffered saline solution containing 0.1% Tween (TBST) for 1 h at room temperature (RT). EGR1 primary antibody (Cell Signaling Technology, EGR1 (44D) Rabbit mAb# 4154, Danvers, MA, USA) was incubated with the membrane overnight at 4 °C in 5% BSA (Bovine Serum Albumin; Fisher Scientific, BP1600-100, Hampton, NH, USA) in TBST. The following day, the membrane was washed for 5 min with TBST for a total of 3 washes. Anti-rabbit secondary antibody was prepared in 5% BSA in TBST and the membrane was incubated with the secondary for 1 h at RT. After the 1 h incubation, the membrane was washed for 5 min with TBST for a total of 3 washes. SuperSignal West Femto Maximum Sensitivity Substrate (Thermo Fisher Scientific, 34096, Waltham, MA, USA) was added to the membrane and subsequently imaged using a ChemiDoc XRS System (Bio-Rad, Hercules, CA, USA).

### 2.5. Transfections

U87MG cells were plated at 7.5 × 10^4^ cells per well in 12-well plates. The following day, cells were transfected with SMARTpool siRNA (Horizon, D-006526-01-005, Lafayette, CO, USA) targeting EGR1 or AllStar negative-control siRNA (Qiagen, 1027280, Germantown, MD, USA) using Dharmafect 1 transfection reagent (Horizon, T-2001-01, Lafayette, CO, USA) per the manufacturer’s instructions. The following day, the culture media was replenished with fresh complete medium and incubated for an additional 48 h prior to infection with virus as described above. Sixteen hours post-infection, cell lysates were collected and either used for Western blot analysis or RNA extraction followed by RT-qPCR. 

### 2.6. Generation of EGR1−/− U87MG Cells

An EGR1−/− U87MG cell pool was generated by Synthego (Menlo Park, CA). U87MG cells were electroporated with Cas9 and a guide RNA specific to EGR1 Exon 1 (Table 1), selected for the presence of Cas9, then shipped to the Dinman lab for further screening. Clonal cell lines were isolated and screened for mutations to EGR1 Exon 1. To isolate clonal cell lines, cells from the knockout pool were seeded at low density in large tissue culture plates and allowed to expand into individual cell colonies. Colony selection was carried out by overlaying the plate with 2% agarose in DMEM then transferring individual, well-isolated colonies to flasks for expansion. Clonal cell lines were assayed for mutations to EGR1 using the Surveyor mutation detection kit from IDT (Cat #706020). Genomic DNA was extracted from potential knockout and wildtype U87MG cells using the Zymo quick-gDNA microprep kit (Zymo Research D3021) and EGR1 Exon 1 was PCR amplified. The PCR products were hybridized with wildtype EGR1 Exon 1 DNA then digested with Surveyor nuclease, per manufacturer’s protocol. EGR1−/− cell lines were expected to yield two digest products, whereas cell lines with intact EGR1 exon 1 would not be digested by the nuclease. Cell lines containing mutations to Exon 1 were further validated using Western blotting.

### 2.7. RNA Isolation and RT-qPCR

Total RNA was extracted using Qiagen’s RNeasy Mini Kit (Qiagen, 7106, Germantown, MD, USA) per the manufacturer’s instructions. Following RNA isolation, samples were quantified using a NanoDrop Spectrophotometer (Thermo Fisher Scientific, ND-ONEC-W, Waltham, MA, USA) and all samples were normalized to 10 ng/µL. Normalized samples were prepared for reverse transcription quantitative PCR (RT-qPCR) using a TaqMan™ RNA-to-Ct 1-step kit (Thermo Fisher Scientific, 4392938, Waltham, MA, USA) per the manufacturer’s instructions. RT-qPCR experiments were performed using the StepOnePlus™ Real-Time PCR System (Thermo Fisher Scientific, 437660, Waltham, MA, USA). TaqMan™ gene expression assays were used to determine changes in gene expression between mock and virally infected samples. Fold changes were calculated relative to 18S ribosomal RNA and normalized to mock samples using the ΔΔCt method. 

### 2.8. Plaque Assays

Extracellular supernatants of virally infected cells were collected at the indicated timepoints and stored at −80 °C. Viral titers were determined by plaque assay in Vero cells as previously described [27]. 

### 2.9. Drug Treatments and CellTiter-Glo Assays

Celecoxib (SelleckChem, S1261, Houston, TX, USA) was diluted in DMSO to a concentration of 100 mM and stored at −20 °C in single-use aliquots. For cytotoxicity experiments, drug was serially diluted 2-fold in cell culture media. Cells were pre-treated with drug for 1 h prior to infection with VEEV. Reserved drug was added back to cells after a 1 h incubation with viral inoculum and two PBS washes. Viability was measured 24 h post-infection using CellTiter-Glo (Promega, G7570, Madison, WI, USA) assays as per the manufacturer’s instructions. Luminescence was measured using a Promega GloMax Discover (Promega, GM3000, Madison, WI, USA) plate reader. 

### 2.10. Statistics

Unless otherwise noted, all statistical analysis was calculated using an unpaired, two-tailed Student’s t-test using Graphpad’s QuickCalcs software. All graphs contain the mean and standard deviations with an n = 3 unless otherwise mentioned.

## 3. Results

### 3.1. Identification of Differentially Expressed Genes (DEGs) Associated with Neuronal Cell Death, Inflammation, or Encephalitis and Consistent with EGR1 Upregulation in VEEV-Infected Cells

**Figure 1:**
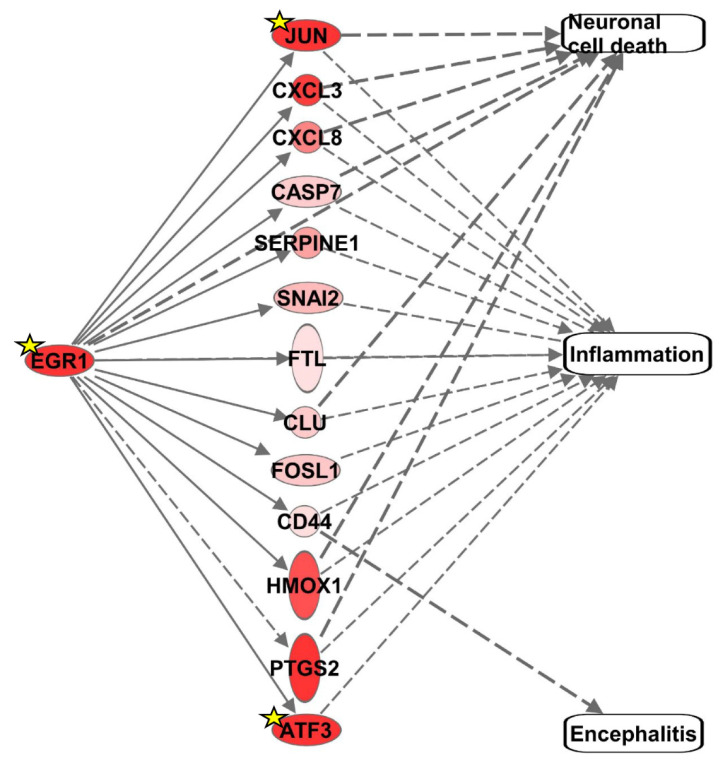
Differentially expressed genes consistent with neuronal cell death, inflammation, encephalitis, and/or EGR1 upregulation. Upregulated genes are displayed in shades of red. The darker the shade, the greater the differential gene expression. Genes with a yellow star contain predicted EGR1 binding sites within their promoter.

In an effort to gain insight into genes that are differentially regulated through EGR1 activation or repression, our RNA-seq data from VEEV-infected human U87MG astrocytoma cells [5] was leveraged. Ingenuity Pathway Analysis (IPA) was used to identify the DEGs following VEEV infection that were consistent with EGR1 activation, focusing on the DEGs involved in neuronal cell death, inflammation, and encephalitis. This analysis identified 14 significant DEGs, including EGR1 (Figure 1 and Table 2). JUN, CXCL3, CXCL8, CASP7, SERPINE1, SNAI2, FLT, CLU, FOSL1, CD44, HMOX1, PTGS2, and ATF3 are all associated with inflammation. JUN, CXCL3, CXCL8, CASP7, EGR1, CLU, HMOX1, and PTGS2 have been linked to neuronal cell death and CD44 is the only DEG consistent with EGR1 activation that is associated with encephalitis. To determine if these genes may be directly modulated by EGR1, the TRANSFAC promoter analysis software was used to generate a list of genes that have predicted EGR1 binding site(s) based on them containing the EGR1 consensus site (GCGG/TGGGCG) ±1 kb of their transcriptional start sites. Only JUN, EGR1, and ATF3 had a predicted EGR1 binding site, suggesting that most of these genes may be indirectly influenced by EGR1. 

### 3.2. Gene Expression of Multiple Inflammatory Mediators and Transcription Factors Are Dependent on EGR1 following VEEV Infection

We next sought to experimentally identify genes that are transcriptionally regulated by EGR1 during VEEV infection. Eight inflammatory mediators were chosen for analysis (Table 3), which included CASP7, CXCL3, CXCL8, and PTGS2 selected by IPA (Figure 1). We also selected TNF-α, CXCL10, and TGF-β due to their link with EGR1, VEEV infection, inflammation, and/or apoptosis. TNF-α is typically upregulated by encephalitic viruses, including alphaviruses [28,29], and has been linked to EGR1 gene regulation [30,31]. Importantly, the TNF-α protein was predicted by IPA to be the most over-represented upstream regulator in our VEEV-infected RNA-seq data. The inflammatory gene CXCL10 was chosen as it has been identified as being differentially upregulated in the brain in vivo following VEEV infection [32]. TGF-β was chosen because it contains an EGR1 binding site, is known to be activated following viral infection, and its expression mediates protection from excessive pathology during acute viral infections [33]. Additionally, four transcription factors were chosen for analysis, including ATF3 and JUN, both selected by IPA, as well as FOS and KLF4 as additional genes of interest (Table 4). FOS was chosen as it forms a heterodimeric complex with JUN, both being members of the AP1 family of transcription factors [34], while KLF4 has been validated to be a transcriptional target of EGR1 [35]. Additionally, FOS has been identified as being differentially upregulated in the brain in vivo following VEEV infection [36].

**Figure 2:**
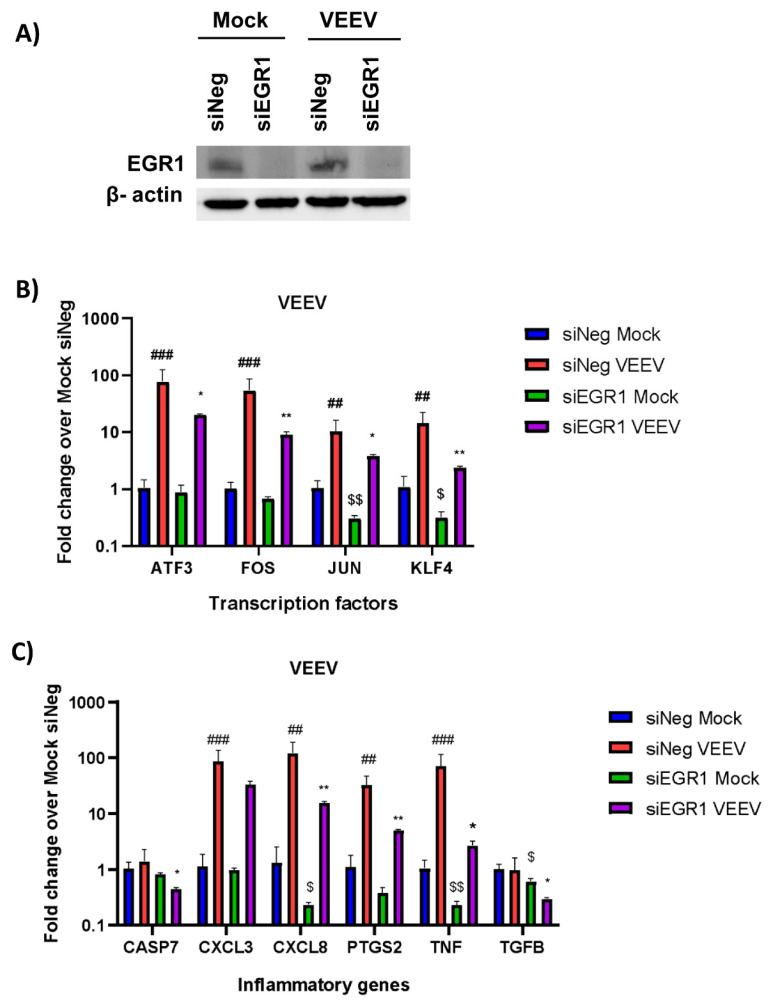
Transcription factors ATF3, FOS, JUN, and KLF4 and inflammatory genes CXCL8, PTGS2, and TNF-α transcription are partially dependent on EGR1 following VEEV infection after knockdown of EGR1 with siRNA. (**A**) U87MG cells were transfected with siNeg or siEGR1 for 48 h prior to infection with VEEV. Knockdown of EGR1 was confirmed via Western blot with β-actin used as the loading control. (**B**,**C**) U87MG cells were transfected in triplicate with either siNeg or siEGR1 for 48 h and then infected with VEEV TrD at an MOI of 5 for 1 h. RNA was extracted from samples collected at 16 hpi. Gene expression for transcription factors (**B**) and inflammatory genes (**C**) was determined by RT-qPCR using TaqMan assays. Data were normalized to mock-infected cells and 18S RNA by the ∆∆CT method. ## *p*-Value ≤ 0.01, ### *p*-Value ≤ 0.001 (comparison between mock siNeg and VEEV siNeg), * *p*-Value < 0.05, ** *p*-Value ≤ 0.01, (comparison between VEEV siNeg and VEEV siEGR1), $ *p*-Value < 0.05, $$ *p*-Value ≤ 0.01 (comparison between mock siNeg and mock siEGR1).

We used two different approaches to examine the EGR1-dependent gene expression following VEEV infection: (1) small interfering RNA (siRNA) to knockdown EGR1 expression in astrocytes and (2) astrocytes engineered via CRISPR/Cas9 to be EGR1-deficient. For the siRNA experiments, the U87MG human astrocytoma cells were treated with an siRNA-targeting EGR1 (siEGR1) or a negative control siRNA (siNeg), and the 72 h post-transfection cells were either mock or VEEV infected at a multiplicity of infection (MOI) of 5. At 16 h post-infection (hpi), the cells were lysed and the knockdown of EGR1 was confirmed via Western blot (Figure 2A). Next, the gene expression was assayed by RT-qPCR in the presence or absence of VEEV TrD infection (Figure 2B,C). The transcription factors ATF3, FOS, JUN, and KLF4 were all found to be significantly upregulated post-VEEV infection and were all partially transcriptionally dependent on EGR1 in this experimental system (Figure 2B). The inflammatory mediators CXCL3, CXCL8, PTGS2, and TNF-α were found to be significantly upregulated following VEEV infection, with CXCL8, PTGS2, and TNF-α identified as being at least partially transcriptionally dependent on EGR1. CASP7 and TGFβ were not significantly altered following VEEV infection. 

**Figure 3:**
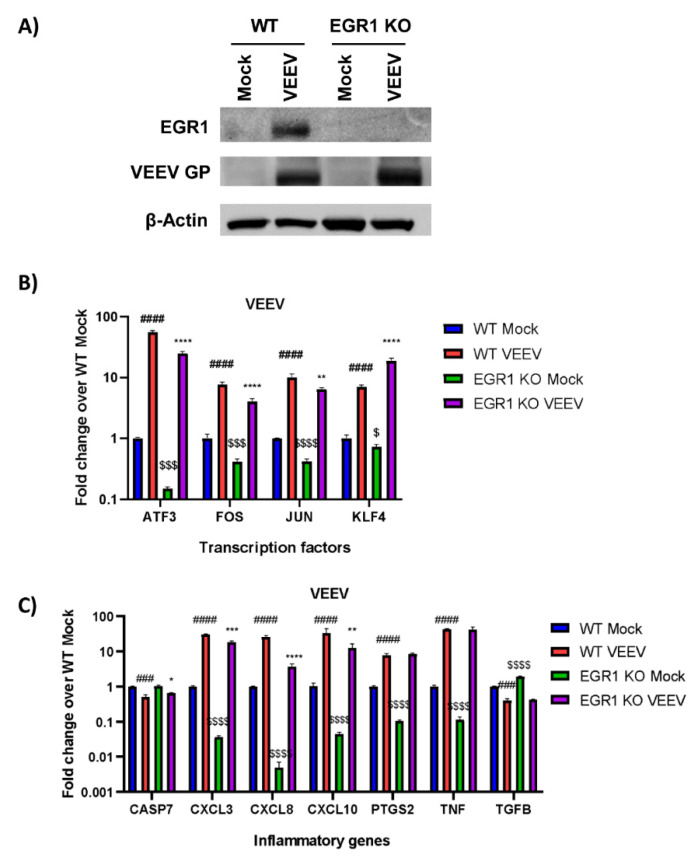
Increased transcription of transcription factors ATF3, FOS, and JUN, as well as inflammatory genes CXCL3 and CXCL8, are at least partially dependent on EGR1 following VEEV infection in EGR1 knockout U87MG cells. (**A**) U87MG WT or EGR1−/−cells were infected with VEEV TC-83 at an MOI of 5 for 1 h. Lysates were collected at 16h post-infection and Western blot analysis performed for EGR1, VEEV GP, and actin. (**B**,**C**) U87MG WT or EGR1−/− cells were infected with VEEV TrD at an MOI of 5 for 1 h. RNA was extracted from samples collected at 16 hpi. Gene expression for transcription factors (**B**) and inflammatory genes (**C**) was determined by RT-qPCR using TaqMan assays. Data were normalized to mock-infected cells and 18S RNA by the ∆∆CT method. ### *p*-Value ≤ 0.001, #### *p*-Value ≤ 0.0001 (comparison between WT mock and WT VEEV), * *p*-Value < 0.05, ** *p*-Value ≤ 0.01, *** *p*-Value ≤ 0.001, **** *p*-Value ≤ 0.0001 (comparison between WT VEEV and EGR1 −/− VEEV), $ *p*-Value < 0.05, $$$ *p*-Value ≤ 0.001, $$$$ *p*-Value ≤ 0.0001 (comparison between WT mock and EGR1−/− mock).

The same panel of genes were assessed for EGR1 transcriptional dependence using a second model system, U87MG cells lacking EGR1 which were generated using CRISPR/Cas9 technology. We aimed to generate the EGR1−/− U87MG cells in order to bypass the use of siRNA transfection as well as to enable a complete loss of EGR1 expression versus a transient knockdown achieved with siRNA. These cells were confirmed to be EGR1 null (EGR1−/−) via western blot analysis (Figure 3A). The WT U87MG cells or EGR1−/− U87MG cells were either mock or VEEV infected and cell lysates were collected 16 hpi. The expression of the transcription factors ATF3, FOS, JUN, and KLF4 were all significantly upregulated following VEEV infection and ATF3, FOS, and JUN were at least partially transcriptionally dependent on EGR1 (Figure 3B). The transcriptional levels of the inflammatory mediator genes were also determined and CXCL3, CXCL8, CXCL10, PTGS2, and TNF-α were significantly upregulated after VEEV infection (Figure 3C). However, only CXCL3, CXCL8, and CXCL10 were identified as being at least partially transcriptionally dependent on EGR1 in this experimental system. Similar to results observed using the siRNA experimental system, changes in CASP7 and TGFβ were minimal and as such they were excluded from further analysis. 

Collectively, these results suggest that multiple inflammatory cytokines/chemokines (TNF-α, CXCL3, CXCL8, CXCL10, PTGS2) are upregulated in VEEV-infected cells in an EGR1-dependent manner in at least one or more experimental systems. These results also demonstrate that EGR1 regulates the transcription of other stress-induced transcription factors (ATF3, FOS, JUN, KLF4) which is relatively conserved between both experimental systems tested. 

### 3.3. EGR Family Members Are Regulated by EGR1

**Figure 4:**
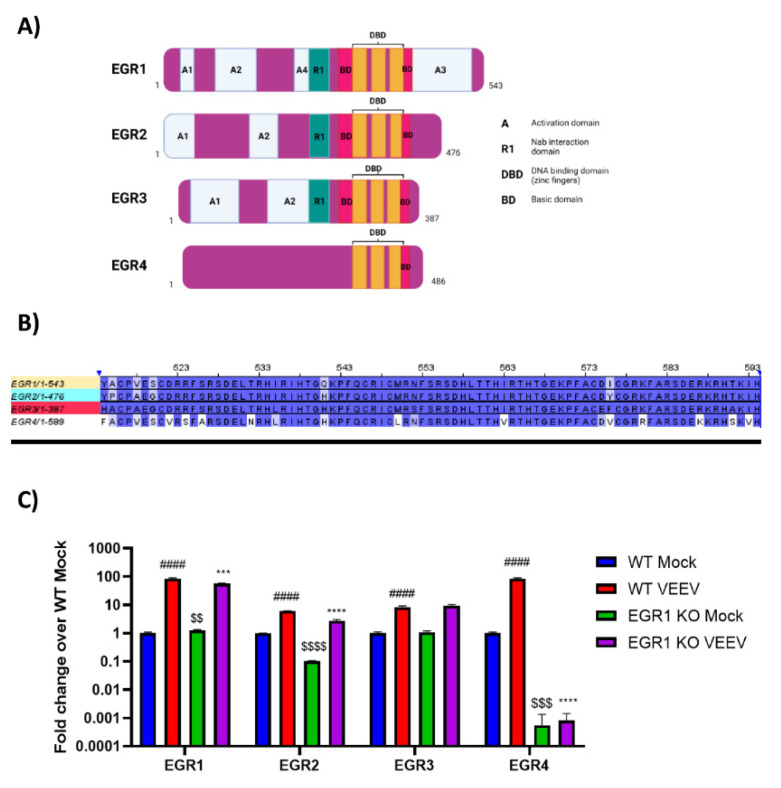
EGR family members are at least partially regulated by EGR1. (**A**) Schematic of EGR family members gene regions. Figure adapted from [49]. (**B**) Alignment of EGR family member DNA binding domains. (**C**) Wildtype or EGR1−/− U87MG cells were infected with VEEV TrD at an MOI of 5 for 1 h. RNA was extracted from samples collected at 16 hpi. Gene expression was determined by RT-qPCR using TaqMan assays. Data were normalized to mock-infected cells and 18S RNA by the ∆∆CT method. #### *p*-Value ≤ 0.0001 (comparison between WT mock and WT VEEV), *** *p*-Value ≤ 0.001, **** *p*-Value ≤ 0.0001 (comparison between WT VEEV and EGR1−/− VEEV), $$ *p*-Value ≤ 0.01, $$$ *p*-Value ≤ 0.001, $$$$ *p*-Value ≤ 0.0001 (comparison between WT mock and EGR1−/− mock).

EGR1 is one member of the early growth response family, which also includes EGR2, EGR3, and EGR4 (Figure 4A, adapted from [49]). The functional DNA binding domain is especially conserved between all four family members (Figure 4B), suggesting the possibility of the compensatory induction of other family members in lieu of EGR1. Therefore, we next assessed if the EGR family members were upregulated following VEEV infection and if the loss of EGR1 had an impact on the transcription of other EGR family members. Gene expression changes were examined in the WT U87MG cells and EGR1−/− U87MG cells. EGR1, EGR2, EGR3, and EGR4 were all upregulated in the VEEV-infected astrocytes (Figure 4C). The detection of EGR1 transcripts in the EGR1−/− U87MG cells is not entirely unexpected as the gene expression assay, which targets the exon 1–2 boundary, may be able to detect mutant transcripts that have not been directed to the Nonsense Mediated Decay (NMD) pathway [50]. The loss of EGR1 significantly suppressed the expression of EGR1 (~28-fold change), EGR2 (~3-fold change), and EGR4 (~84-fold change) transcription in the VEEV-infected cells, but EGR4 transcription was the most dramatically impacted family member, with the EGR4 transcripts nearly at the limit of detection (Supplemental Table S1). These results suggest that EGR1 regulates, at least partially, the expression of other EGR family members following VEEV infection. 

### 3.4. EGR1 Is Upregulated in VEEV-, EEEV-, SINV-, CHIKV-, ZIKV-, and RVFV-Infected Cells

**Figure 5:**
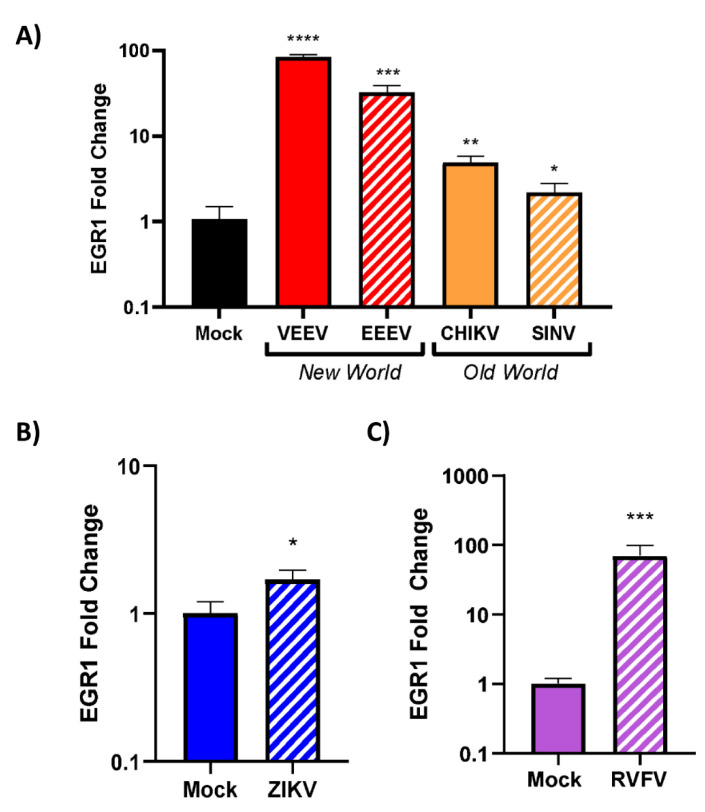
EGR1 is upregulated in VEEV-, EEEV-, SINV-, CHIKV-, ZIKV-, and RVFV-infected cells. WT U87MG cells were seeded at 1 × 10^5^ cells/well. The following day, cells were infected with alphaviruses, including (**A**) VEEV, EEEV, SINV, or CHIKV, (**B**) ZIKV, a flavivirus, or (**C**) RVFV, a phlebovirus, at an MOI of 5 in serum-free media for 1 h. After 1 h incubation, cells were washed 2× with PBS and replenished with fresh serum-free media. Cell lysates were collected 16 hpi and RNA was extracted and normalized to 10 ng/uL. Gene expression was measured using TaqMan assays for EGR1 and 18s. Data were normalized to 18s and mock was set to 1. *—indicates significance as compared to mock, * *p*-value < 0.05, ** *p*-value < 0.007, *** *p*-value < 0.0005, **** *p*-value < 0.0001.

We next sought to assess whether EGR1 upregulation is limited to VEEV infection or if EGR1 is globally upregulated following viral infections in general. Multiple viruses that are known to replicate within the CNS were chosen for this analysis [51]. This included an additional New World alphavirus (EEEV), two Old World alphaviruses (CHIKV and SINV), a flavivirus (ZIKV), and a phlebovirus (RVFV) (Table 5). EEEV infection resulted in a ~30-fold increase in EGR1 expression as compared to mock-infected cells (Figure 5A). CHIKV and SINV were examined due to their association with encephalitis (although rare). The results indicated that both CHIKV and SINV infection results in an increase (~5-fold over mock and ~2-fold over mock, respectively) in EGR1 expression post-infection, albeit milder as compared to New World alphaviruses (Figure 5A). EGR1 was slightly (~1.5-fold over mock), but significantly, upregulated in response to ZIKV infection (Figure 5B). Finally, EGR1 was dramatically upregulated (~70-fold over mock) in response to infection with RVFV (Figure 5C), a phlebovirus known to induce major damage to the CNS [52]. These results suggest that EGR1 induction could be a global host response to infection with RNA viruses in U87MG cells. 

### 3.5. Loss of EGR1 Has Minimal Impact on VEEV, EEEV, CHIKV, SINV, RVFV, and ZIKV Viral Titers

**Figure 6:**
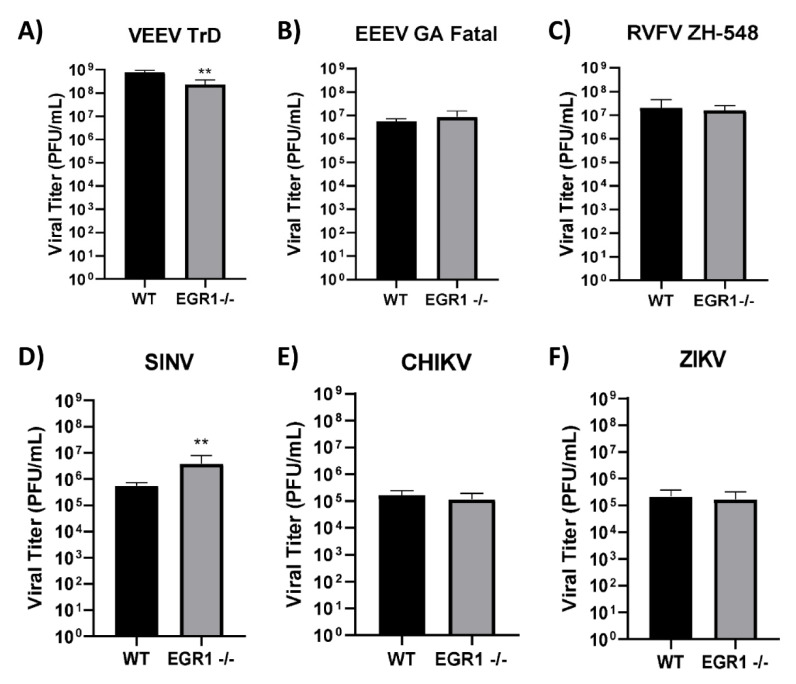
Loss of EGR1 has minimal impact on VEEV, EEEV, CHIKV, SINV, RVFV, and ZIKV viral titers. WT U87MG or EGR1−/− U87MG cells were seeded at 1 × 10^5^ cells/well. The following day, cells were infected with either (**A**) VEEV, (**B**) EEEV, (**C**) RVFV, (**D**) SINV, (**E**) CHIKV, or (**F**) ZIKV at an MOI 5 in serum-free media for 1 h. After 1 h incubation, cells were washed 2× with PBS and replenished with fresh serum-free media. At 16 hpi, the supernatant was collected, and viral titers were determined via plaque assay. ** *p*-value: 0.0011 (VEEV); 0.0082 (SINV).

Because EGR1 is strongly upregulated in VEEV, EEEV, and RVFV infections, we next sought to see if the loss of EGR1 had any impact on infectious viral titers as compared to wildtype U87MG cells. To this end, we infected EGR1−/−U87MG cells in addition to WT U87MG cells with virus (VEEV, EEEV, RVFV, SINV, CHIKV, and ZIKV) at an MOI of 5 and collected cellular supernatants at 16 hpi. Infectious viral titers were determined via plaque assay (Figure 6A–F). The loss of EGR1 had no significant impact on viral replication in EEEV, RVFV, CHIKV, and ZIKV. Conversely, the VEEV titers were reduced by ~0.5log_10_ in the EGR1-deficient cells, while the SINV titers were significantly increased in the EGR1−/− U87MG cells (Figure 6D), with a difference in the viral titers of ~0.85log_10_. These results indicate that the loss of EGR1 has little to no impact on the VEEV, EEEV, RVFV, SINV, CHIKV, and ZIKV production in human astrocytes. 

### 3.6. EGR1-Dependent Gene Expression in EEEV-, SINV-, CHIKV-, ZIKV-, and RVFV-Infected Cells

Because EGR1 is significantly upregulated following infection with EEEV, SINV, CHIKV, ZIKV, and RVFV and because all of these viruses are known to induce encephalitis (Table 5), we next sought to examine EGR1-dependent gene expression in these viruses. To this end, the WT or EGR1−/− U87MG cells were mock or virally infected at an MOI of 5 for 1 h. The RNA was extracted from samples collected at 16 hpi. The gene expression for the same transcription factors and inflammatory mediator genes as above was determined by RT-qPCR using TaqMan assays. 

**Figure 7:**
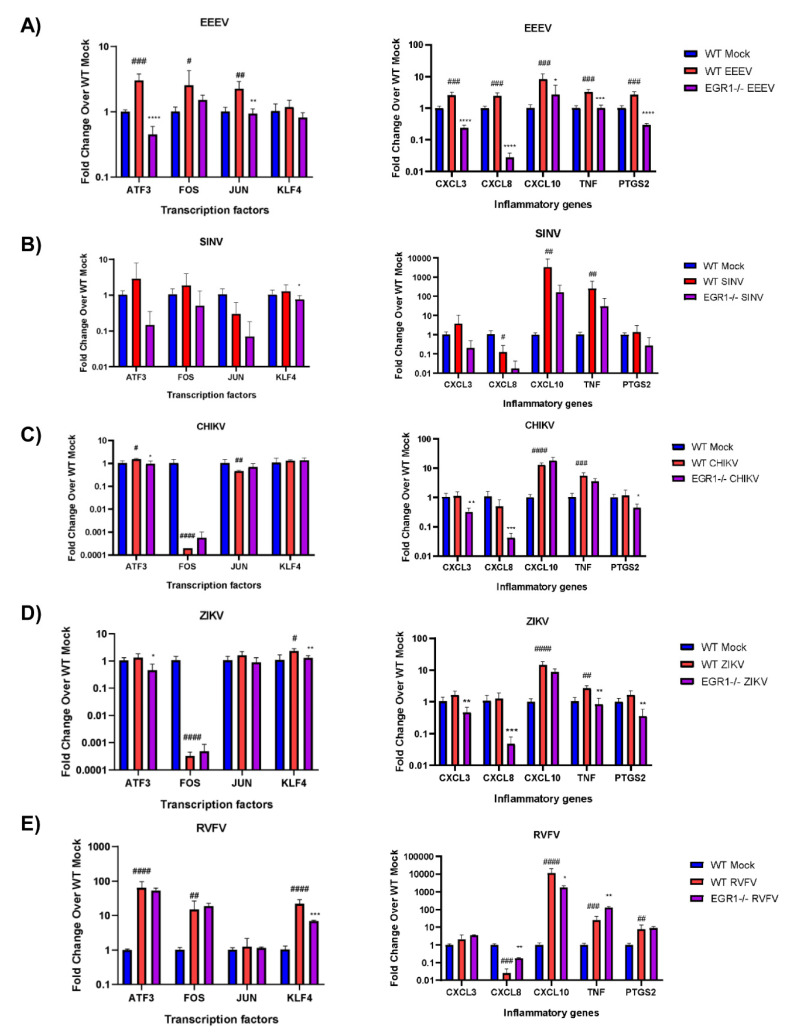
EGR1-dependent gene expression in EEEV-, SINV-, CHIKV-, ZIKV-, and RVFV-infected cells. WT and EGR1−/− U87MG cells were infected with either mock, (**A**) EEEV, (**B**) SINV, (**C**) CHIKV, (**D**) ZIKV, or (**E**) RVFV at an MOI of 5 for 1 h. RNA was extracted from samples collected 16 hpi. Gene expression for transcription factors and inflammatory genes was determined by RT-qPCR using TaqMan assays. Data were normalized to mock-infected cells and 18S RNA by the ∆∆CT method. # *p*-Value < 0.05, ## *p*-Value ≤ 0.01, ### *p*-Value ≤ 0.001, #### *p*-Value ≤ 0.0001 (comparison between WT mock and WT VEEV), * *p*-Value < 0.05, ** *p*-Value ≤ 0.01, *** *p*-Value ≤ 0.001, **** *p*-Value ≤ 0.0001 (comparison between WT VEEV and EGR1−/− VEEV).

Infection of the astrocytoma cells with EEEV resulted in a significant upregulation of transcription factors ATF3, FOS, and JUN, with both ATF3 and JUN being at least partially transcriptionally dependent on EGR1 (Figure 7A). Interestingly, following EEEV infection, all of the inflammatory genes tested, including CXCL3, CXCL8, CXCL10, TNF, and PTGS2, were significantly upregulated and all were identified as being at least partially transcriptionally dependent on EGR1. Importantly, the EGR1 dependency observed following EEEV infection correlates with the results observed during VEEV infection, suggesting that EGR1 regulation of these genes may be conserved amongst New World alphavirus family members.

We next examined CHIKV and SINV, both Old World alphaviruses, for EGR1-dependent gene expression post-infection. Of the transcription factors assayed, only KLF4 was identified as being at least partially transcriptionally dependent on EGR1 in the SINV-infected cells (Figure 7B). The inflammatory genes CXCL10 and TNF were significantly upregulated following SINV infection, and while levels of expression for both were reduced in the EGR1−/− infected cells, neither were statistically significant. Infection of astrocytoma cells with CHIKV resulted in a slight, but significant, increase in ATF3 transcription (Figure 7C). Meanwhile, FOS and JUN were both significantly downregulated following CHIKV infection but were not impacted by the loss of EGR1. The inflammatory genes CXCL10 and TNF were both significantly upregulated following CHIKV infection, but not in an EGR1-dependent manner. Interestingly, the loss of EGR1 resulted in a significantly decreased expression of CXCL3, CXCL8, and PTGS2 as compared to WT-infected cells. 

Infection with ZIKV resulted in a significant increase in KLF4 expression as well as a dramatic decrease in FOS transcription (Figure 7D). ATF3 transcription was slightly, but not significantly, upregulated following ZIKV infection in WT cells but transcription was significantly reduced in the EGR1 null-infected cells. Additionally, KLF4 transcription was at least partially dependent on EGR1. Of the inflammatory genes assayed, CXCL10 and TNF-α were significantly upregulated at 16 hpi with ZIKV, and while both were reduced in the EGR1−/− infected cells, only TNF-α was reduced to statistically significant levels and identified as transcriptionally dependent on EGR1. CXCL3, CXCL8, and PTGS2 were all slightly, but not significantly, induced following ZIKV infection, and the expression of all three inflammatory mediators was significantly decreased in the EGR1−/− infected cells as compared to the WT mock-infected cells. 

Finally, the expression of ATF3, FOS, and KLF4 was markedly increased following RVFV infection, with only KLF4 observed as being at least partially transcriptionally dependent on EGR1 (Figure 7E). The transcription of JUN was unaffected between both cell types following infection. CXCL10, TNF-α, and PTGS2 were all significantly upregulated following RVFV infection, with CXCL10 transcription identified as being at least partially dependent on EGR1. Interestingly, the loss of EGR1 resulted in an even more robust increase in TNF-α transcription. CXCL8 expression was significantly decreased following infection, and the loss of EGR1 resulted in an increase in CXCL8 transcription. 

Collectively, these results indicate that EGR1 induction following viral infection at least partially regulates transcription factors and inflammatory mediators, including ATF3 (EEEV, CHIKV, ZIKV), JUN (EEEV), KLF4 (SINV, ZIKV, RVFV), CXCL3 (EEEV, CHIKV, ZIKV), CXCL8 (EEEV, CHIKV, ZIKV, RVFV), CXCL10 (EEEV, RVFV), TNF-α (EEEV, ZIKV, RVFV), and PTGS2 (EEEV, CHIKV, ZIKV). 

### 3.7. Inhibition of PTGS2 with Celecoxib Rescues Cells from VEEV-Induced Cells Death but has No Effect on Viral Titers

**Figure 8:**
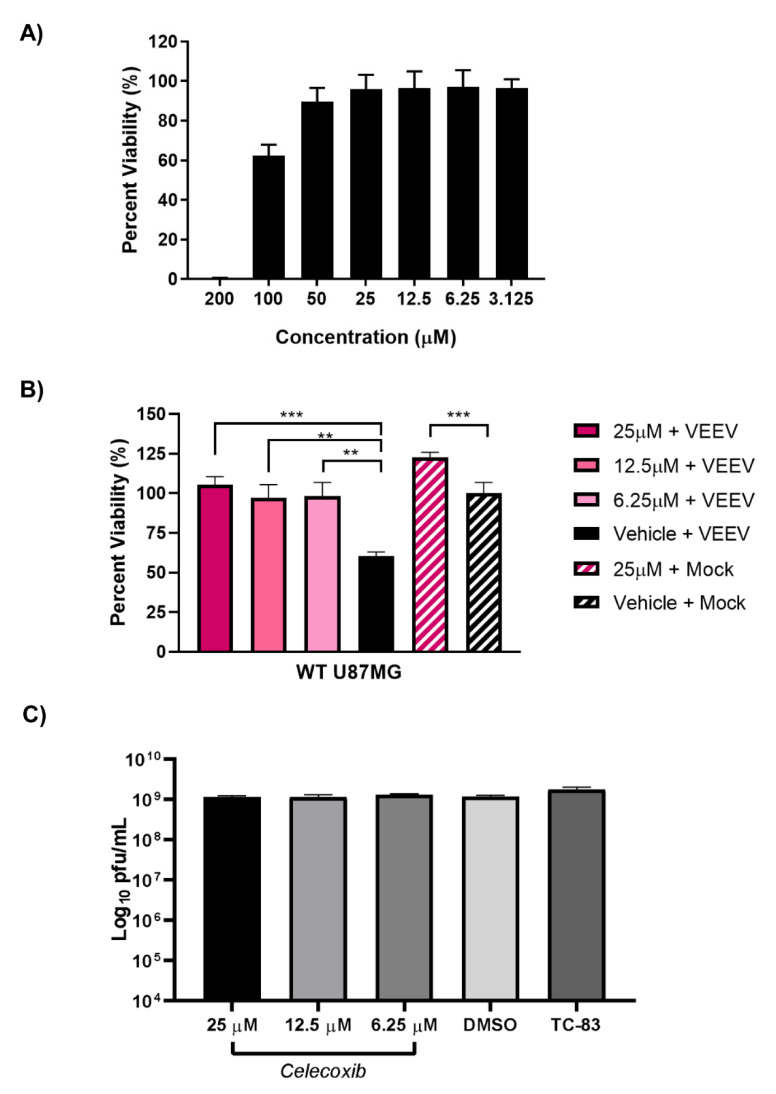
Inhibition of PTGS2 with Celecoxib rescues cells from VEEV-induced cell death but has no effect on viral titers. (**A**) U87MG cells were treated with ≤200 µM of celecoxib for 24 h. Cell viability was determined using Cell Titer Glo with mock set to 100%. (**B**) U87MG cells were pre-treated with 25, 12.5, or 6.25 µM of celecoxib or DMSO vehicle control prior to infection with VEEV for 1 h. After 1 h infection, inoculum was removed and washed 2× with PBS prior to treated media being added back to the cells. Viability was determined 24 hpi using Cell Titer Glo with vehicle + mock-infected cells being set to 100%. *** *p*-Value ≤ 0.0001, ** *p*-Value ≤ 0.005. Mock treated vs. mock vehicle *p*-value 0.0005. (**C**) U87MG cells were pre-treated with 25, 12.5, or 6.25 µM of celecoxib or DMSO vehicle control prior to infection with VEEV for 1 h. After 1 h infection, cells were washed 2× with PBS prior to treated media being added back to cells. Supernatant was collected 16 hpi and viral titers determined via plaque assay.

Prostaglandin-endoperoxide synthase 2 (PTGS2) is a key enzyme involved in prostaglandin biosynthesis associated with physiological stresses, such as infection and inflammation [62,63]. PTGS2 is at least partially dependent on EGR1 during VEEV and EEEV infection and, as such, we next sought to determine if inhibiting PTGS2 with celecoxib, a small molecule inhibitor, could rescue cells from virally induced cell death. As a first step, the toxicity of celecoxib was determined empirically by measuring the cell viability after treatment with ≤200 µM in the U87MG cells (Figure 8A). Concentrations 25 µM and below were non-toxic and, as such, we chose to move forward with 25 µM. Wildtype U87MG cells were pre-treated for 1 h with either 25, 12.5, or 6.25 µM of celecoxib or vehicle control prior to infection with VEEV for 1 h at an MOI of 5. Following infection, the viral inoculum was removed, and the cells were washed twice with PBS to remove residual virus. After washing, the reserved treated media was added back to the cells, and after 24 h, the cell viability was assessed. The Celecoxib-treated cells were partially rescued from VEEV-induced cell death as compared to the vehicle-treated infected cells (Figure 8B). The Celecoxib-treated cells ranged from 97–105% viability, whereas only 60% viability was observed with the vehicle-treated VEEV-infected cells. The statistical analysis of the vehicle-treated mock-infected cells compared to the celecoxib-treated mock-infected cells indicated that the celecoxib treatment enhanced the growth of the mock-infected cells. However, the difference in the cell viability of the VEEV-infected cells treated with celecoxib (~45%) is greater than that observed in the mock-infected cells treated with celecoxib (~25%). This suggests that the impact of celecoxib is not solely due to enhancing cell growth, but rather is also preventing VEEV-induced cell death. The cellular supernatants were collected to assess differences in the viral titers between the treated and untreated infected cells; however, no significant differences in the viral titers were observed (Figure 8C). Altogether, these results suggest that inhibition of PTGS2, one downstream target of EGR1, partially alleviates VEEV-induced apoptosis without impacting viral titers. 

## 4. Discussion

The transcriptomic analysis and mining of RNA-sequencing data combined with IPA enabled us to bioinformatically identify the DEGs consistent with EGR1 upregulation and known involvement in neuronal cell death, inflammation, and/or encephalitis. Genes matching these criteria, as well as other genes of interest, were further examined using multiple in vitro experimental systems in order to determine the influence of EGR1 on their transcriptional activation or repression following VEEV infection. VEEV infection induced robust upregulation of CXCL3, CXCL8, PTGS2, and TNF-α at 16 hpi. The knockdown of EGR1 with siRNA enabled us to identify inflammatory mediators CXCL8, PTGS2, and TNF-α as being at least partially transcriptionally dependent on EGR1. Additionally, stress-induced transcription factors ATF3, FOS, JUN, and KLF4 were all significantly upregulated at 16 hpi with VEEV and were also all identified as being at least partially transcriptionally regulated by EGR1. To examine the consequences of the complete loss of the functional EGR1 protein, a second experimental system using astrocytes engineered through CRISPR/Cas9 to be deficient of EGR1 was employed. In this experimental system, inflammatory mediators CXCL3, CXCL8, CXCL10, PTGS2, and TNF-α were robustly induced following VEEV infection, with CXCL3, CXCL8, and CXCL10 identified as being at least partially transcriptionally dependent on EGR1. Likewise, transcription factors ATF3, FOS, JUN, and KLF4 were all significantly upregulated following VEEV infection and were all identified as being at least partially transcriptionally dependent on EGR1. Of the transcription factors examined, ATF3 has been shown by others to be critical for producing infectious virus in vitro [64]. Similarly, both FOS and JUN have been shown to be differentially upregulated in the brain in vivo following VEEV infection [32,36]. KLF4 is upregulated following VEEV infection and has previously been implicated as a negative regulator of host innate immune response antiviral signaling [65]. Interestingly, KLF4 transcription in VEEV-infected cells was only dependent on EGR1 under the siRNA-mediated knockdown of EGR1 conditions. KLF4 and EGR1 together have been implicated in inducing ATF3 transcription and subsequent apoptosis in resveratrol-treated human colorectal cancer cells [66]. While both KLF4 and EGR1 are transcription factors known to regulate cell fate decisions, most of these interactions have only been confirmed in vitro and thus remain to be verified in vivo [9,67]. Regardless, all of the transcription factors examined here require further studies to identify the underlying mechanism of how EGR1 is, at least partially, controlling their induction following VEEV infection as well as their roles in VEEV-induced apoptosis. In terms of inflammatory mediators, CXCL10 has been confirmed to be differentially expressed in the brains of mice infected with VEEV [32]. Importantly, CXCL10 activity has been attributed to neuronal apoptosis following encephalitic arboviral infection [68]. CXCL3 has been shown to be significantly upregulated after VEEV infection in vitro and in vivo [64,69]. Additionally, the knockdown of CXCL3 with siRNA in vitro resulted in decreased virus production [64]. TNF-α is upregulated following VEEV infection and studies involving TNF receptor knockout mice resulted in extended survival times [70]. Additionally, the brains of VEEV-infected mice lacking TNF receptors suffered from neurodegeneration even in regions in which VEE antigen staining was negative and thus further corroborating the role of inflammatory mediators in inducing neuronal insults following VEEV infection [70]. 

The differences in the results obtained between the two different systems presented here is an obvious limitation of these studies. However, both experimental systems come with inherent variability. While siRNA is widely used in research, the knockdown mechanism it imposes on target mRNA is transient and the complete loss of protein expression is not achievable. Conversely, complete knockout through CRISPR/Cas9 of a protein critically involved in neurological development and stress-induced cellular responses can induce compensatory effects within the cell. The differences observed between the two systems in our studies propelled us to look deeper into the other EGR family members and their dependence on EGR1 following VEEV infection. All four EGR family members were found to be significantly upregulated in VEEV-infected astrocytoma cells. However, only EGR1, EGR2, and EGR4 were identified as being at least partially transcriptionally dependent on EGR1. Expression of EGR3 was subtly, but not significantly, increased in VEEV-infected EGR1−/− cells as compared to WT-infected cells. In addition, the loss of EGR1 did not reduce EGR2 expression levels to those observed in mock-infected cells. Thus, it is possible that either EGR2 or EGR3 may be partially compensating for the loss of EGR1 during VEEV infection. All four of the EGR family members are immediate early genes and are readily expressed in the brain. Importantly, all four members share a highly conserved zinc-finger DNA binding domain (DBD) that preferentially interacts with a conserved GC-rich promoter sequence GCGC(G/T)GGCG, known as the EGR response element (ERE) [49]. Due to their highly homologous DBDs, it is possible the EGR family members overlap in their respective targets and function [6]. Conversely, the N-terminal regions differ considerably between all four members, suggesting specificity in protein–protein interactions and therefore potential regulatory and functional differences between family members [6,49]. We fully acknowledge that EGR1 likely does not work in isolation and other factors may contribute to its changes in transcriptional regulation. Thus, future studies will focus on combinatorial knockdown of the EGR family members in an effort to determine if and how other EGR family members are compensating for the loss of EGR1.

We also examined the EGR1 gene dependency in other viruses known to induce encephalitis. The most striking EGR1 dependency was observed in EEEV-infected astrocytoma cells. Following EEEV infection, transcription factors ATF3 and JUN and inflammatory genes CXCL3, CXCL8, CXCL10, TNF-α, and PTGS2 were all at least partially transcriptionally dependent on EGR1. EEEV is the least well-studied of the encephalitic alphaviruses and, as such, the current literature lacks insight as to changes occurring at the cellular level in the host following infection with EEEV. More studies are required to further elucidate the underlying host–pathogen interactions and whether EGR1 mediates inflammatory genes that are critically involved in the host’s antiviral response. In addition, further studies will determine if EGR1 exerts similar effects on the other EGR family members during infection with other viruses known to induce encephalitis, such as RVFV.

CHIKV infection in astrocytoma cells produced a robust induction of CXCL10 and TNF-α, two inflammatory genes well documented for their role in enhancing the severity of disease in human cases as well as in mouse models of infection [71,72]. However, neither CXCL10 or TNF-α transcription was dependent on EGR1 in this cell type and timepoint assayed. Nevertheless, EGR1 has been identified as a direct upstream regulator of neutrophil activation following CHIKV infection and CXCL3 was a predicted target of EGR1 per IPA [73]. CXCL3 and CXCL8 were identified as being at least partially transcriptionally dependent on EGR1 in our dataset; however, neither were upregulated in WT CHIKV-infected cells at 16 hpi which may be too early of a timepoint as both CXCL3 and CXCL8 have been documented to be significantly upregulated in human primary fibroblasts at 24 hpi [74]. Infection of astrocytoma cells with SINV, another Old World alphavirus, significantly induced transcription of CXCL10 and TNF-α, but neither were dependent on EGR1. Similar to CHIKV, both CXCL10 and TNF-α contribute to SINV-induced pathogenesis and overt pathology, but most in vitro and in vivo studies examine SINV-induced effects at 24 hpi or later timepoints [75,76]. 

The results from the ZIKV-infected astrocytoma cells were very similar to the results observed following CHIKV infection, with both CXCL10 and TNF-α robustly induced following infection. TNF-α was at least partially transcriptionally dependent on EGR1. The transcription factor KLF4 was also significantly induced post-infection and also at least partially transcriptionally dependent on EGR1. While not significantly upregulated following infection, transcription of ATF3, CXCL3, CXCL8, and PTGS2 was at least partially dependent on EGR1. PTGS2 and TNF have both been implicated as being overexpressed during ZIKV infection, with TNF overexpression being directly associated with severe microcephaly [77]. Thus, it would be interesting to further explore the role of EGR1 in regulating PTGS2 and TNF-α expression following ZIKV infection. 

Infection with RVFV induced significant upregulation of ATF3, FOS, KLF4, CXCL10, TNF-α, and PTGS2 in astrocytoma cells at 16 hpi. KLF4 and CXCL10 were identified as being at least partially transcriptionally dependent on EGR1. CXCL8 expression was significantly decreased following infection and was at least partially dependent on EGR1. Both CXCL10 and CXCL8 have been implicated as biomarkers in human cases of RVFV with hemorrhagic manifestations [78]. EGR1 has been implicated as playing pro-apoptotic and pro-inflammatory roles in mouse models of RVFV infection [79]. As such, further examination of EGR1′s influence on gene expression following RVFV infection in vivo would be interesting to explore. 

Celecoxib, a small molecule inhibitor targeting PTGS2, was examined for its ability to prevent VEEV-induced cell death. Clinical applications for celecoxib as an anti-inflammatory, analgesic, and/or antipyretic medication have been utilized for well over two decades [80]. The allure of celecoxib over traditional NSAIDs is attributed to the safer gastro-intestinal profile; however, controversy exists surrounding its consumption and increased risk of heart attacks [81]. In our study, the celecoxib treatment rescued astrocytes from VEEV-induced cell death but had no significant effect on infectious viral titers. Others have tested celecoxib against VEEV in vitro and have observed similar anti-inflammatory effects [82]. Furthermore, treatment of cells with celecoxib prior to VEEV infection seemingly alleviated the induction of TNF-α in microglial cells [82]. Inhibiting the activity of PTGS2 reduces VEEV-induced pro-inflammatory cytokine production which likely contributes to cell survival. While celecoxib may not target the virus directly, its anti-inflammatory effects have the potential to alleviate VEEV-induced neuronal insults and could be utilized in a combinatorial treatment approach. 

**Figure 9:**
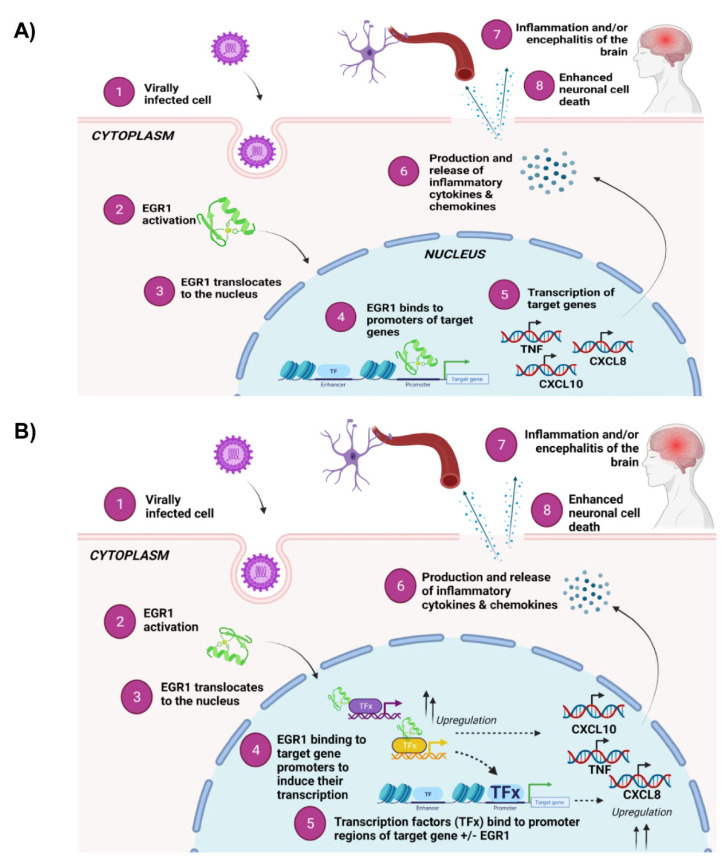
EGR1 may work directly or indirectly with target genes to induce transcription of inflammatory cytokines and chemokines and apoptotic transcriptional machinery. (**A**) Overview of proposed direct mechanism of action. Susceptible cells become virally infected, resulting in EGR1 activation and translocation to the nucleus. Once in the nucleus, EGR1 binds directly to promoters of target genes and induces their transcription, thereby contributing to inflammation and cell death associated with viral infection. (**B**) Overview of proposed indirect mechanism of action. Susceptible cells become virally infected, resulting in EGR1 activation and translocation to the nucleus. Once in the nucleus, EGR1 binds to target genes, such as transcription factor x, resulting in their upregulation. Transcription factor(s) X then binds to promoter regions of inflammatory genes and apoptotic machinery, inducing their transcription and subsequent release of inflammatory cytokines and chemokines, ultimately contributing to neuronal cell death within the brain. Figures were created with BioRender.com.

Finally, EGR1 may directly and/or indirectly impact gene expression after viral infection. We propose that EGR1 may directly bind the promoter region of target genes to directly influence their transcription (Figure 9A). Alternatively, or perhaps alongside, EGR1 may indirectly regulate gene expression by interacting with proteins that are already occupying the promoter region of target genes to influence their transcription (Figure 9B). Regardless of how EGR1 is influencing gene expression, ultimately, the release of inflammatory cytokines and chemokines are detrimental to the CNS. Further, what is specifically triggering these neuronal insults? Is it a consequence of viral infection and therefore virally mediated? Or is it a consequence of the host inflammatory response being activated and stressed to a point where it is no longer repairable? Studies have suggested that the host immune response is activated following viral infection, and for some neurotropic viruses, such as VEEV, the accompanying fatal encephalitis is often a consequence of the host’s frenzied immune response to active viral replication in the brain and not a direct consequence of viral infection alone [32,83]. Further studies are required to validate our proposed mechanism of action as well as to tease out the underlying processes into more detail. Nevertheless, EGR1 plays a complex role in a diverse set of biological functions and systems. While EGR1 is most notably regarded for its involvement in brain development, plasticity, learning, and memory, it is actively being explored in other areas of research, including its role in viral infections. The role of EGR1 in learning and memory combined with its dramatic upregulation following VEEV infection makes EGR1 an attractive research target because VEEV is a neurotropic and neurovirulent virus known to induce pathological lesions in the brain. Additionally, because children are at a higher risk of developing encephalitis, it is important that we elucidate the mechanisms underlying VEEV-induced neuronal insults in order to develop therapeutic interventions. Identifying and understanding changes in gene expression at the transcriptional level in response to viral infection can serve to identify potential target(s) for vaccine and therapeutic development. 

## Figures and Tables

**Table 1:** Oligonucleotides used for generation of EGR1−/− U87MG cells.

Description	Sequence
Guide RNA target sequence-located in Exon 1 of EGR1	5′ ctttcctcactcgcccacca 3′
EGR1 Exon 1 Fwd	5′ gagagatccagccgcagaac 3′
EGR1 Exon 1 Rev	5′ cggtcaggtgctcgtaggg 3′

**Table 2:** Differentially expressed genes consistent with neuronal cell death, inflammation, encephalitis, and/or EGR1 upregulation.

Gene	Entrez Gene Name	Log_2_ Fold Change ^1^	*p*-Value ^1^	FDR *p*-Value ^1^
JUN	Jun proto-oncogene, AP-1 transcription factor subunit	3.510	2.67 × 10^−97^	3.37 × 10^−94^
CXCL3	C-X-C motif chemokine ligand 3	3.225	7.30 × 10^−4^	0.03
CXCL8	C-X-C motif chemokine ligand 8	2.556	0.02	0.39
CASP7	Caspase 7	1.269	0.03	0.47
SERPINE1	Serpin family E member 1	2.170	1.21 × 10^−90^	1.41 × 10^−87^
SNAI2	Snail family transcriptional repressor 2	1.740	2.05 × 10^−4^	9.67 × 10^−3^
FTL	Ferritin Light Chain	0.556	5.35 × 10^−5^	3.29 × 10^−3^
CLU	Clusterin	1.316	7.56 × 10^−11^	1.52 × 10^−8^
FOSL1	FOS-like 1, AP-1 transcription factor subunit	1.411	3.9 × 10^−9^	6.02 × 10^−7^
CD44	CD44 Molecule	0.651	5.86 × 10^−4^	0.02
HMOX1	Heme oxygenase 1	3.076	3.58 × 10^−39^	2.37 × 10^−36^
PTGS2	Prostaglandin-endoperoxide synthase 2	5.525	4.79 × 10^−7^	5.37 × 10^−5^
ATF3	Activating transcription factor 3	5.260	2.36 × 10^−40^	1.64 × 10^−37^
EGR1	Early growth response 1	5.841	1.58 × 10^−81^	1.56 × 10^−78^

^1^ Values are from [5].

**Table 3:** Inflammatory genes chosen for further analysis.

Gene	Entrez Gene Name	Function	Log_2_ Fold Change ^1^
CASP7	Caspase 7	Involved in the activation cascade of caspases responsible for apoptosis execution [37].	1.269
CXCL3	C-X-C Motif Chemokine Ligand 3	Chemokine involved in inflammation; chemoattractant for neutrophils [38].	3.225
CXCL8	C-X-C Motif Chemokine Ligand 8	Major mediator of the inflammatory response. Functions as a chemotactic factor by guiding the neutrophils to the site of infection [39,40].	2.556
CXCL10	C-X-C Motif Chemokine Ligand 10	Stimulates production of monocytes, natural killer and T-cell migration, and modulation of adhesion molecule expression [41].	N/A *
TNF-α	Tumor Necrosis Factor Alpha	Pro-inflammatory cytokine involved in regulation of a wide variety of biological processes, including apoptosis [42].	7.415
PTGS2	Prostaglandin-Endoperoxide Synthase 2	Key enzyme in prostaglandin biosynthesis: a group of lipids made at sites of tissue damage or infection that are involved in dealing with injury and illness [43].	5.525
TGF-β	Transforming growth factor beta (TGF-β)	Multifunctional cytokine. Plays a role in immune and stem cell regulation and differentiation [44].	0.447

^1^ Values are from [5]. * CXCL10 was not found in the dataset from [5].

**Table 4:** Transcription factors chosen for further analysis.

Gene	Entrez Gene Name	Function	Log_2_ Fold Change ^1^
ATF3	Activating transcription factor 3	Binds the cAMP response element (CRE) (consensus: 5′-GTGACGT[AC][AG]-3′), a sequence present in many viral and cellular promoters. Plays a role in regulating the cell cycle and apoptosis [45].	5.260
FOS	Fos Proto-Oncogene	Dimerizes with proteins of the JUN family, thereby forming the transcription factor complex AP-1. Involved in regulation of cell proliferation, differentiation, transformation, and apoptosis [46].	3.387
JUN	Jun Proto-Oncogene	Second factor of AP-1 transcription factor complex. Interacts with specific target DNA sequences to regulate gene expression [47].	3.510
KLF4	Kruppel-Like Factor 4	Thought to control the G1-to-S transition of the cell cycle following DNA damage by mediating the tumor suppressor gene p53 [48].	4.360

^1^ Values are from [5].

**Table 5:** Viruses utilized to elucidate the influence of EGR1 in other encephalitic viral infections.

Virus	Family, Genus	Known to Cause Encephalitis?
Eastern equine encephalitis virus	*Togaviridae, Alphavirus* (New World)	Yes [53,54,55]
Chikungunya virus	*Togaviridae, Alphavirus* (Old World)	Sometimes [56,57,58,59]
Sindbis virus	*Togaviridae, Alphavirus* (Old World)	Sometimes [56,60]
Zika virus	*Flaviviridae, Flavivirus*	Yes [57,58,59]
Rift Valley fever virus	*Phenuiviridae, Phlebovirus*	Yes [52,59,61]

## Data Availability

Data are contained within the article.

## Supplementary Materials

The following supporting information can be downloaded at: https://www.mdpi.com/article/10.3390/v14061210/s1, Table S1: Ct values of EGR family member analysis.
